# Understanding the ligamentum teres of the hip: a histological study

**DOI:** 10.1590/1413-78522015230101030

**Published:** 2015

**Authors:** Bryan Wang Dehao, Tan Kong Bing, James Loh Sir Young

**Affiliations:** I Changi General Hospital, Department of Orthopedic Surgery, Singapore, Department of Orthopedic Surgery, Changi General Hospital, Singapore; II National University Hospital, Department of Pathology, Singapore, Department of Pathology, National University Hospital, Singapore

**Keywords:** Ligament, Hip, Science

## Abstract

**Objective:**

To perform a histological study describing the microstructure of the ligamentum teres of the hip, with special emphasis on the presence of nerve bundles. Our study aims to correlate the microstructure of the ligamentum teres with its postulated functions, allowing greater understanding of its role within the hip joint.

**Methods:**

Fresh specimens excised intra-operatively in 11 patients undergoing open hip procedures were preserved in formalin and sent to the laboratory for histological analysis by our collaborating pathologist. The specimens were sectioned and stained, and examined under the microscope to look at their microstructure. In addition, a novel staining technique was employed to detect neural elements within the individual specimens .

**Results:**

The ligamentum teres is composed predominantly of a connective tissue matrix of collagen fibers, fibrous and adipose tissue, with an overlying layer of investing synovium. In addition, there are blood vessels of various sizes surrounded by a layer of encircling fat. In all specimens examined, there were nerve bundles of various shapes and sizes, regardless of the age of the patient.

**Conclusion:**

The ligamentum teres has both mechanical and biological functions within the hip joint and should no longer be considered a developmental vestige. Where possible, any surgical procedures around the hip joint should aim to limit damage to this structure to minimize any potential loss of function. Level of Evidence Basic Science Study.

## INTRODUCTION

There have been several studies looking into the role of the ligamentum teres of the hip since the 19th century,^1^ yet little has been elucidated as to its true function, with many considering it to be predominantly a vestigial structure in adult hips. However, with the increasing use of hip arthroscopy, there has been an increased interest in the microstructure, function and pathology of the ligamentum teres. What was once thought to be an essentially functionless structure has been found to play a role in stabilization of the hip joint,^2^ and also as a potential contributory factor in patients with persistent hip pain.^3^ We are also beginning to appreciate its importance in pediatric hip stability, and techniques have been described to preserve this structure in procedures involving open reduction of dislocated hips where possible.^4^

Current data suggests mechanical as well as biological functions of the ligamentum teres within the hip joint, although corroborative data in human studies remains fairly limited. Mechanically, it is believed that the ligamentum teres may function as a checkrein to prevent excessive abnormal motion of the hip joint. In a cadaveric study performed by Demange et al, he found that there was a slight increase in the mean adduction of the hip following arthroscopic sectioning of the ligamentum teres,^2^ suggesting a role in stability and constraint of the hip joint. Histological studies done in animal studies have shown that the distribution of collagen in the ligamentum teres of the hip is similar to that of the collateral and cruciate ligaments of the knee,5 indicating a possible role as a static stabilizer within the hip joint in view of their similar properties. There have also been case reports of recurrent subluxation of the hip joint in athletes following injuries or tears to the ligamentum teres,6 again suggesting a possible role as a checkrein within the hip joint.

Biologically, the ligamentum teres is thought to provide some vascularity to the adult femoral head, although this supply is believed to be limited and variable. Also, there have been studies to support the theory that pathologies involving the ligamentum teres do contribute to nociception of the hip joint. In fact, studies have shown that lesions involving the ligamentum teres are the third most common source of hip pain in athletes who undergo a diagnostic arthroscopic procedure.^3^ Also, arthroscopy performed in painful hips without diagnosis has revealed structural damage such as partial/complete tears or degeneration of the ligament,^7^ suggesting a role of the ligamentum teres in joint nociception. Partial and complete ruptures of the ligamentum teres of the hip can lead to both instability symptoms as well as persistent hip pain,^3^ and arthroscopic debridement of isolated ruptures have resulted in symptomatic improvement in more than 80% of cases.^8^

The aims of this study are firstly to perform a histological analysis of the ligamentum teres to describe its overall microstructure, with special emphasis on the presence of any neural elements through a novel staining technique, and secondly, to correlate these findings with its postulated functions.

This study is the first known study looking at the microstructure of the ligamentum teres using fresh human specimens excised intra-operatively, and we hope that our findings will provide us with a greater understanding of the postulated mechanical and biological functions of the ligamentum teres within the hip joint.

## MATERIALS AND METHODS

This is a histological study looking at fresh specimens of ligamentum teres excised intra-operatively. This study was made possible by an intra-hospital research grant following approval by the hospital research committee. We also applied for ethics clearance and approval by our cluster's Institutional Review Board.

Specimens were excised intra-operatively by a senior orthopedic surgeon from 12 patients, with a mean age of 71 (range 36 to 86) years old, undergoing hip surgery for various reasons. (Table 1) We excluded patients who had severe osteoarthritis or other severe inflammatory arthropathies. Specimen from one patient had to be discarded due to inadequate preservation in formaldehyde solution, leaving us with a total of 11 specimens.

**Table 1.** Demographic data.

Case #	Age (years old)	Gender	Diagnosis
1	36	M	Femoral neck fracture
2	84	F	Femoral neck fracture
3	83	F	Femoral neck fracture
4	68	F	Femoral neck fracture
5	86	F	Femoral neck fracture
6	53	F	Osteoarthritis
7	77	M	Femoral neck fracture
8	65	F	Osteoarthritis
9	84	F	Femoral neck fracture
10	70	F	Femoral neck fracture
11	80	F	Osteoarthritis

The ligamentum teres was sharply dissected from the femoral head and the acetabular notch. To allowed the pathologist to identify the orientation of the specimen correctly, the acetabular end was marked with a suture. The specimens were then fixed in 4% formaldehyde solution and subsequently brought down to the pathology laboratory for further preparation and analysis of the specimens.

Following preparation of the specimens, they underwent general processing first to assess their general microstructure and architecture, followed by special immunohistochemistry processing to look for neurofilaments in order to identify the presence of nerve bundles.

### Histopathology Protocol

### General processing

Formalin-fixed ligamentum teres specimens were received by the pathologist. They were subsequently described and measured. They were thereafter serially sectioned at 3mm intervals from the femoral aspect to the acetabular aspect. Tissue processing into paraffin blocks was carried out. Tissue sections at 4µm thickness were cut and stained with the standard Haematoxylin and Eosin (H+E) method and mounted.

### Immunohistochemistry processing

To better visualize the neural tissue, coated slides with tissue sections were stained using the Roche Ventana BenchMark XT System^(r)^. Retrieval was done with Cell Conditioning Solution (CC1) Standard Neurofilament (Clone 2F11, DekoCytomation Denmark A/S) diluted to 1:500 and incubated for 32 minutes at 37^o^C. Detection of nerve endings was done using Ventana Ultraview DAB^(r)^ kit. This is a novel multimer-based detection system, which allows the detection of antigens which are present at low concentrations, in this case, the neurofilament protein-expressing nerve bundles. At the end of the immunohistochemistry staining, counter-staining of the nuclei with Haematoxylin for 1 min was carried out. Dehydration, clearing and mounting of the slides with DPX was then performed.

### Microscopic assessment

Examination of the H+E tissue sections was carried out. The overall tissue organization was observed and recorded. For blood vessels, their nature (i.e. small artery, vein, arteriole and venule) and presence of congestion were recorded. The caliber of the largest small artery for each specimen was recorded using an eyepiece micrometer. The diameter of the most sizable nerve bundles was similarly recorded. In addition to the native tissue components, the presence of any general pathological features such as an inflammatory cellular infiltrate, fibrosis or metaplasia was also noticed. Representative histological photographs were taken. The histological findings were summarized in Table 2. 

**Table 2.** Summary of histological findings.

**Synovium**	• Single cuboidal layer • Present in all specimens
**Blood vessels **	• Size of largest arteries (mean 182µm, range 30-400µm) • All small arteries surrounded by layer of encircling adipose fat
**Nerve tissue **	• Present in all specimens regardless of age • Different shapes and sizes • Largest diameter (mean 56.5µm, range 15-100µm)
**Adipose tissue **	• Present in all specimens, with embedded blood vessels • Fat necrosis in those with high-energy trauma
**Collagen, fibrous tissue **	• Similar constituent to knee cruciate ligaments • Present in all specimens
**Others **	• Chondroid and osteochondroid metaplasia • Increased prevalence in those with more severe degenerative changes

## RESULTS

The ligamentum teres of the hip is surrounded by a layer of investing synovium, which is composed of a single layer of cuboidal cells. The underlying connective tissue matrix is made up predominantly of randomly oriented collagen fibers, fibrous and adipose tissue, as well as interspersed blood vessels and nerve bundles.

**Figure 1.**
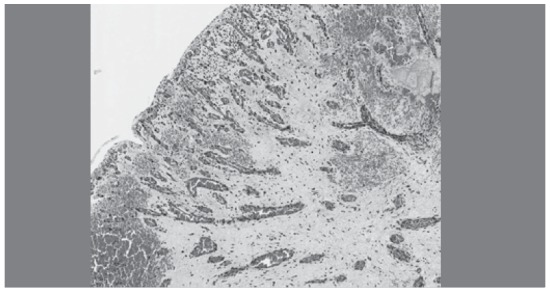
Underlying congestion and hemorrhage.

**Figure 2.**
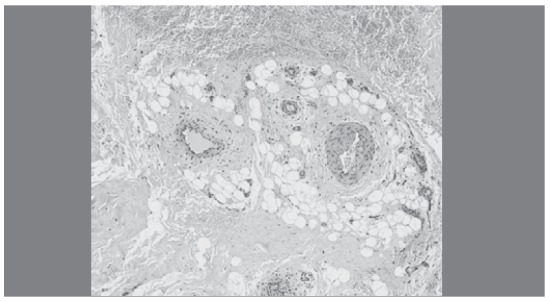
Various sized blood vessels in encircling fat.

In our study, small arteries, veins, arterioles and venules were present in all specimens. The diameters of the largest caliber arteries ranged from 30µm to 400µm (mean 182µm). As expected, significant amounts of congestion and hemorrhage were noticed in specimens coming from patients with hip fractures arising from high-energy trauma, such as motor vehicle accidents. (Figure 1) Smaller amounts of hemorrhage were also noticed in patients with severe osteoarthritis of the hips. Interestingly, it was also noticed that most of the small arteries were surrounded by a layer of encircling fat. (Figure 2)

**Figure 3.**
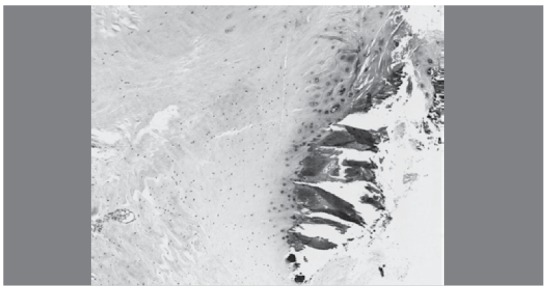
Osteo-chondroid tissue within fibrous stroma.

**Figure 4.**
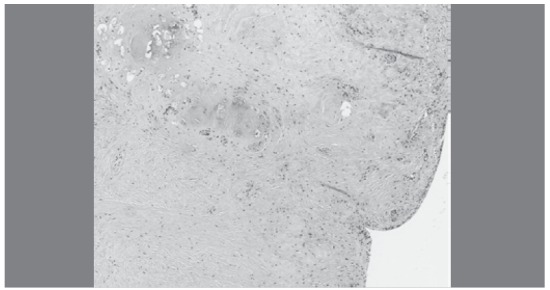
Chondroid tissue within fibrous stroma.

**Figure 5.**
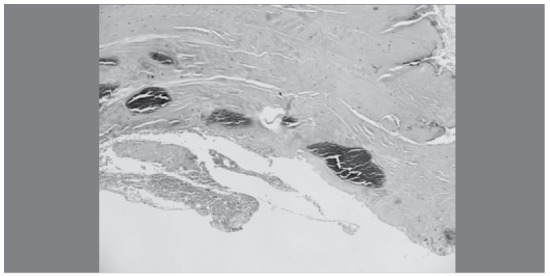
Basophilic crystalline deposits (x100).

**Figure 6.**
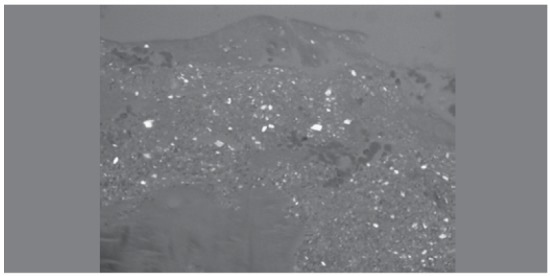
Positively-birefringent crystalline deposits.

There were also specimens which demonstrated features of chronic inflammatory infiltration as well as metaplastic change, specifically, the presence of osteo-chondroid and chondroid tissue within the specimens. (Figures 3 and 4) Whilst these findings were not consistent across the board, they were more prevalent in those patients who had more severe degenerative changes and symptoms of pain. Interestingly, one patient was also found to have positively-birefringent crystals within the ligamentum teres, suggesting calcium pyrophosphate crystal deposition. The patient was otherwise asymptomatic, and did not have any known history or clinical evidence of crystalline arthropathy. (Figures 5 and 6)

**Figure 7.**
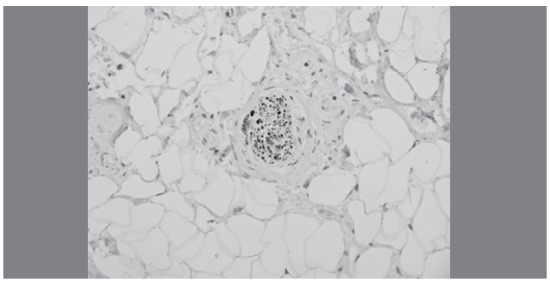
Immunohistochemistry showing presence of nerve bundles.

**Figure 8.**
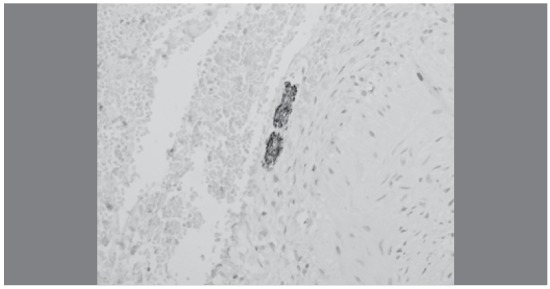
Immunohistochemistry showing presence of nerve bundles.

With the aid of the special immunohistochemistry processing, we were also able to identify the presence of neurofilament protein-expressing nerve bundles, although we were unable to delineate the exact subtype or function of the individual bundles. During histo-analysis, neural elements were found in all specimens examined, regardless of the age of the patient. The nerve bundles identified were of varying shapes and sizes, (Figures 7 and 8) and were scattered throughout the connective tissue layer. The largest diameters of the various nerve bundles were measured and were found to be between the ranges of 15µm to 100µm (mean 56.5µm).

In summary, the ligamentum teres of the hip is composed of a single layer of investing synovium, with an underlying framework of connective tissue. Within the connective tissue, blood vessels, adipose tissue, collagen fibers, and fibrous tissue make up the majority of the components. The blood vessels were of various sizes, and the small arteries were often surrounded by a layer of encircling adipose tissue. Nerve bundles were also found scattered throughout the connective tissue layer in all specimens examined. This was regardless of the age or gender of the patient, and they were also of various shapes and sizes. In some specimens, there was evidence of metaplastic change, either as a result of degeneration or ongoing adaptive processes.

## DISCUSSION

This is the first study looking at the overall microstructure of the ligamentum teres (including analysis of its neural elements) in fresh human specimens, as opposed to previous studies, which have been performed largely in cadaveric or animal studies. Whereas other studies have studied the microstructure of the specimens in isolation from analysis of the neural elements, we have been able to perform a histo-analysis of both components through a special staining techniques employed by our collaborating pathologist. 

Whilst we know from the landmark anatomical study done by Sevitt and Thompson9 that the artery of the ligamentum teres has clearly no function in the vascularization of the adult femoral head, our study has shown that the ligamentum teres is not an entirely avascular structure. In fact, there are blood vessels of various sizes, ranging from small arteries, arterioles, veins and venules. The small arteries were also noticed to be consistently surrounded by a layer of encircling adipose tissue, providing a cushioning effect to maintain the patency of these vessels. As such, these vessels may not be as insignificant as previously considered, and may provide some contribution (however limited) to the vascularity of the femoral head, and also as nutrition conduit for other components within the ligamentum teres itself. 

Also, from our study, we can see that the majority of the connective tissue of the specimens is comprised mainly of collagenous and fibrous tissue, with a constitution similar to the cruciate and collateral ligaments of the knee. This suggests that the ligamentum teres of the hip may serve as a static stabilizer within the hip joint, just as the knee ligaments are the primary static stabilizers of the knee joint. This has also been supported by other cadaveric studies,^2^ which have shown an increase in the range of motion of the hip joint following selective sectioning of the ligamentum teres. Also, patients with arthroscopically diagnosed tears of the ligamentum teres often present with episodes of â€˜giving way' of the hip, or instability symptoms,^10^ again suggesting a possible stabilizing role within the hip joint. 

Interestingly, we also found evidence of metaplastic change in some of the specimens that were examined. There were occasional foci of osteochondroid or chondroid changes within the connective tissue component, and these findings were more prevalent in those who had more severe degenerative changes within the hip joint. As such, the ligamentum teres is a more dynamic structure than previously believed, and may undergo changes in response to a changing external environment. Whether this translates to an increased risk of developing degenerative tears of the ligamentum teres still remains unclear, but it certainly does represent an adaptive response secondary to changes within the hip joint.

More importantly, from our study we can establish that the ligamentum teres of the hip contains nerve bundles of various shapes and sizes within its connective tissue matrix. These were seen in all specimens, regardless the age of the patient. These may include somatosensory afferents which provide sensory feedback to prevent excessive and abnormal motions of the hip joint. As such, these nerve bundles may contribute to proprioception of the hip joint, allowing the ligamentum teres to function essentially as a â€˜checkrein' against excessive movements of the hip joint. These somatosensory afferents may also participate in fine coordination of the hip joint. Clinically, this has been shown in athletes with tears of ligamentum teres of the hip who present with recurrent subluxation of the hip joint,^11^ possibly as a result of loss of fine coordination. 

Moreover, the presence of nerve bundles within the ligamentum teres may suggest a possible role in nociception of the hip joint. This has been postulated through clinical studies whereby hip pain has been reported in patients with ruptured ligamentum teres in otherwise healthy hips.^12^ Also, judicious debridement of a diagnosed partial tear of the ligamentum teres has been found to reduce persistent hip pain in patients undergoing this procedure.^13^

As the adult hip is a fairly well-constrained socket joint, the ligamentum teres may serve as a secondary joint protection system through the activation of a reflex system when the ligamentum teres is stretched out beyond physiological limits. The excessive tensioning of a distracted ligamentum teres may provide afferent signals to inhibit further joint excursion through reflex activation of the muscles surrounding the hip joint. Previous studies have also shown the presence of nerve endings in the acetabular labrum,^14^ as such, they may work in tandem to limit excessive joint motions to minimize damage to the acetabular rim and adjacent cartilage as well.

As such, we can surmise that the ligamentum teres is certainly not a structure without function in the hip joint, and should be treated with more care. Our study has shown that the ligamentum teres is likely to have nervous functions, and it is also a dynamic structure that can adapt and respond to changes in the external environment. (Table 3) This is important in guiding treatment of pathologies involving the ligamentum teres. When treating patients with painful hips secondary to tears of the ligamentum teres, the debridement should remain limited, attempting to preserve as much of it as possible. This is to minimize the impact of loss of the various nerve bundles, which has an important role to play in protection against excessive mobilization of the hip as well as fine coordination. Furthermore, unless these postulated functions can be disproved, the use of surgical procedures which may sacrifice the ligamentum teres of the hip should be carefully reconsidered.

**Table 3.** Postulated functions of the ligamentum teres.

**Mechanical**	**1. Stability ** • Rich in collagen and fibrous stiffness comparable to cruciate ligaments of the knee Able to show adaptive changes in response to external stress **2. Proprioception and fine-coordination** • Nerve bundles present in all specimens, likely including somatosensory afferents contributing to the secondary joint protection system
**Biological**	**1. Vascular supply **• Blood vessels of varying sizes scattered throughout structure Layer of encircling fat around small arteries providing structural support to maintain lumen patency **2. Nociception** • Nerve bundles present in all specimens, likely including pain fibers contributing to hip pain in ligamentum tears or ruptures

However, there is still a need for further studies so that we can have a clearer understanding of the postulated functions of the ligamentum teres of the hip. Further biomechanical studies performed on cadaveric specimens will perhaps be useful to understand how tears or ruptures to the ligamentum teres can result in instability of the hip. However, it is clear from our studies that the ligamentum teres of the hip is certainly more than just an unimportant developmental vestige, and thus, should no longer be considered as such.

This study has, however, some limitations. Firstly, the demographics of our sample population was largely skewed towards the older age group, as it was difficult to attain specimens in younger patients, due to the small number of patients within that age group undergoing hip procedures. Secondly, due to current technical constraints, we were unable to perform a more in-depth analysis of the specimens, particularly with respect to the neural elements. Ideally, newer methods to delineate the exact nature of the nerve tissue (proprioceptive versus nociceptive) should be explored. However, we have aimed to have a greater overview of the specimens that were harvested, including looking at the overall microstructure of the other elements within the ligamentum teres, rather than focusing entirely on the neural elements in isolation.

## CONCLUSION

This is the first known study describing the overall microstructure of the ligamentum teres (and its neural elements) using fresh human specimens. Based on our findings, the ligamentum teres should no longer be considered a developmental vestige, as there is evidence to suggest that it has both mechanical and biological functions within the hip joint. As such, any surgical procedures should aim to minimize excessive damage to this structure as far as possible, so as to minimize any potential loss of function.
